# Resveratrol Supplementation Attenuates Cognitive and Molecular Alterations under Maternal High-Fat Diet Intake: Epigenetic Inheritance over Generations

**DOI:** 10.3390/ijms22031453

**Published:** 2021-02-01

**Authors:** Vanesa Izquierdo, Verónica Palomera-Ávalos, Mercè Pallàs, Christian Griñán-Ferré

**Affiliations:** 1 Department of Pharmacology and Therapeutic Chemistry, Institut de Neurociències—Universitat de Barcelona, Avda. Joan XXIII, 27, 08028 Barcelona, Spain; vanessa_izquierdo@hotmail.com (V.I.); pallas@ub.edu (M.P.); 2 Department of Cellular and Molecular Biology, University Center of Biological and Agricultural Sciences, University of Guadalajara, km 15.5 Guadalajara-Nogales highway, 45110 Zapopan, Jalisco, Mexico; vpalomera@hotmail.com

**Keywords:** cognitive decline, epigenetics, resveratrol, high-fat diet, aging, SAMP8, N^6^-Methyladenosine methylation, multigenerational inheritance

## Abstract

Environmental factors such as maternal high-fat diet (HFD) intake can increase the risk of age-related cognitive decline in adult offspring. Epigenetic mechanisms are a possible link between diet effect and neurodegeneration across generations. Here, we found a significant decrease in triglyceride levels in a high-fat diet with resveratrol (RSV) HFD + RSV group and the offspring. Firstly, we obtained better cognitive performance in HFD+RSV groups and their offspring. Molecularly, a significant increase in DNA methylation (5-mC) levels, as well as increased gene expression of *DNA methyltransferase 1* (*Dnmt1)* and *Dnmt3a* in HFD + RSV F1 group, were found. Furthermore, a significant increase of N^6^-Methyladenosine methylation (m^6^A) levels in HFD+RSV F1, as well as changes in gene expression of its enzymes *Methyltransferase like 3 (Mettl3*) and *FTO alpha-ketoglutarate dependent dioxygenase (Fto)* were found. Moreover, we found a decrease in gene expression levels of pro-inflammatory markers such as *Interleukin 1β (Il1-β), Interleukin 6 (Il-6)*, *Tumor necrosis factor-α (Tnf-α), C-X-C motif* *chemokine ligand 10 (Cxcl-10)*, the pro-inflammatory factors monocyte chemoattractant protein 1 (Mcp-1) and *Tumor growth factor-β1 (Tgf-β1)* in HFD+RSV and HFD+RSV F1 groups. Moreover, there was increased gene expression of neurotrophins such as *Neural growth factor (Ngf*), Neurotrophin-3 (Nt3), and its receptors *Tropomyosin receptor kinase TrkA* and *TrkB*. Likewise, an increase in protein levels of brain-derived neurotrophic factor (BDNF) and phospho-protein kinase B (p-Akt) in HFD+RSV F1 was found. These results suggest that maternal RSV supplementation under HFD intake prevents cognitive decline in senescence-accelerated mice prone 8 (SAMP8) adult offspring, promoting a reduction in triglycerides and leptin plasma levels, changes in the pro-inflammatory profile, and restoring the epigenetic landscape as well as synaptic plasticity.

## 1. Introduction

Due to the advancing life expectancy in modern societies, research in aging-hallmarks leading to neurodegeneration and Alzheimer’s disease (AD), is an important scientific field [1]. Aging has been linked to progressive brain deterioration, leading to impaired cognitive function, and increasing vulnerability to death [2]. This cognitive impairment is commonly accompanied by different molecular alterations like inflammation, synaptic dysfunction, and epigenetic modification, among others [3]. Therefore, pleiotropic compounds to rescue them is a significant field of research [4].

In this regard, nutrition and supplementation are important components of healthy brain aging, particularly in those with dementia [5]. It is well-established that the consumption of a high-fat diet (HFD) increases the risk of several chronic diseases, including age-related cognitive decline [6]. Accordingly, studies have evidenced the link between HFD and neurodegeneration through several altered pathways. Among them, Wang et. al. (2020) found the inflammation and synaptic plasticity [7]. Inflammation and synaptic dysfunction are two of the most important events in neurodegeneration and AD; further inflammation can alter synaptic plasticity engendering cognitive impairment [8,9]. Microglia activation is considered the main hallmark of neuroinflammation that releases pro-inflammatory cytokines such as Interleukin 1 beta (IL1-ß), Interleukin 6 (IL-6), or Tumor necrosis factor-α (TNF-α) [10]. Likewise, the synaptic plasticity is primarily mediated by neurotrophins, among them, brain-derived neurotrophic factor (BDNF), and neurotrophin-3 (NT3), allowing the permissive conditions under which plasticity can appear [11,12].

On the other hand, evidence suggests that epigenetic mechanisms are an important contributor to the pathogenesis of age-related neurodegenerative diseases [13,14]. Likewise, there is a relationship between the effect of neuroinflammation, synaptic dysfunction and the epigenetic landscape [15]. Recently, it has been shown that nutrients can affect epigenetic marks such as DNA methylation (5-mC) and hydroxymethylation (5-hmC) as well as RNA N^6^-methyladenosine methylation (m^6^A), thereby promoting changes in gene expression of critical genes associated with the pathophysiology of several age-related diseases, including AD [16,17]. Thus, epigenetics has emerged as a tool for understanding a broad range of human diseases, such as type 2 diabetes mellitus, obesity, inflammation, and neurodegenerative disorders [14,18,19]. In utero, an HFD supplementation in rodents causes a metabolic syndrome-like (MeS) state that can be transmitted across multiple generations from directly exposed generation (transgenerational inheritance—F1) to indirectly exposed generation (transgenerational inheritance—F2) through non-genetic factors such as epigenetic mechanisms [20,21]. Moreover, several studies have demonstrated that HFD exposure promoted leptin’s epigenetic modifications, persisting for multiple generations [22,23,24]. Thus, transgenerational epigenetic inheritance can be modulated by exposures occurring during life in the P0 generation, impacting in the F2, as the F1 germline cells have not been directly exposed. Overall, those key events demonstrate the active involvement in the maintenance of neuronal integrity, synapses and cognitive function for the individual and their offspring [25,26].

Resveratrol (RSV) (3,5,4′-trihydroxy-trans-stilbene) is a natural phytoalexin produced in grapes, peanuts, and their derivatives, with a plethora of beneficial effects as an anti-inflammatory and synaptic plasticity inductor, including epigenetic modifications [27,28]. RSV also exerts neuroprotection in several neuropathological conditions [29,30,31]. Previous results from our group have demonstrated that RSV modulates key pathways for correct neuronal function, improving cognitive decline in the senescence-accelerated mouse prone 8 (SAMP8) and its offspring [32]. However, the pleiotropic mechanism of action of RSV is not completely described, although increasing evidence in animal models brings to light the beneficial effects of polyphenols such as RSV across generations [33,34].

The SAMP8 mouse is a well-established aging animal model, generated by phenotypic selection from the genetic pool of AKR/J mice [35]. The SAMP8 strain manifests behavioral alterations, age-related cognitive decline, as well as neuropathological AD hallmarks [36]. Jointly with AD hallmarks, several key events associated with neurodegeneration are presented in SAMP8 mice such as neuroinflammation [35], and synaptic dysfunction [13,37]. Of note, those changes are accompanied by epigenetic alterations [17,38]. Furthermore, several studies have demonstrated that non-pharmacological interventions such as environmental enrichment (EE), and diet supplementation can induce changes in the epigenome, reducing cognitive decline and modifying key neurodegenerative pathways altered in SAMP8 [39,40].

Concretely, the present work aims to prove the neuroprotective effects of maternal RSV supplementation under HFD intervention in SAMP8 offspring through neuroinflammation and synaptic dysfunction as well as delving deep into the epigenetic patterns modulated by a RSV supplementation. Therefore, we demonstrated that SAMP8 under HFD enriched with RSV showed a preserved cognitive decline across generations, being an important insight for RSV use in public health interventions favoring healthy aging.

## 2. Results

### 2.1. Body Weight Progression in SAMP8 HFD + RSV and Its Offspring

**Figure 1:**
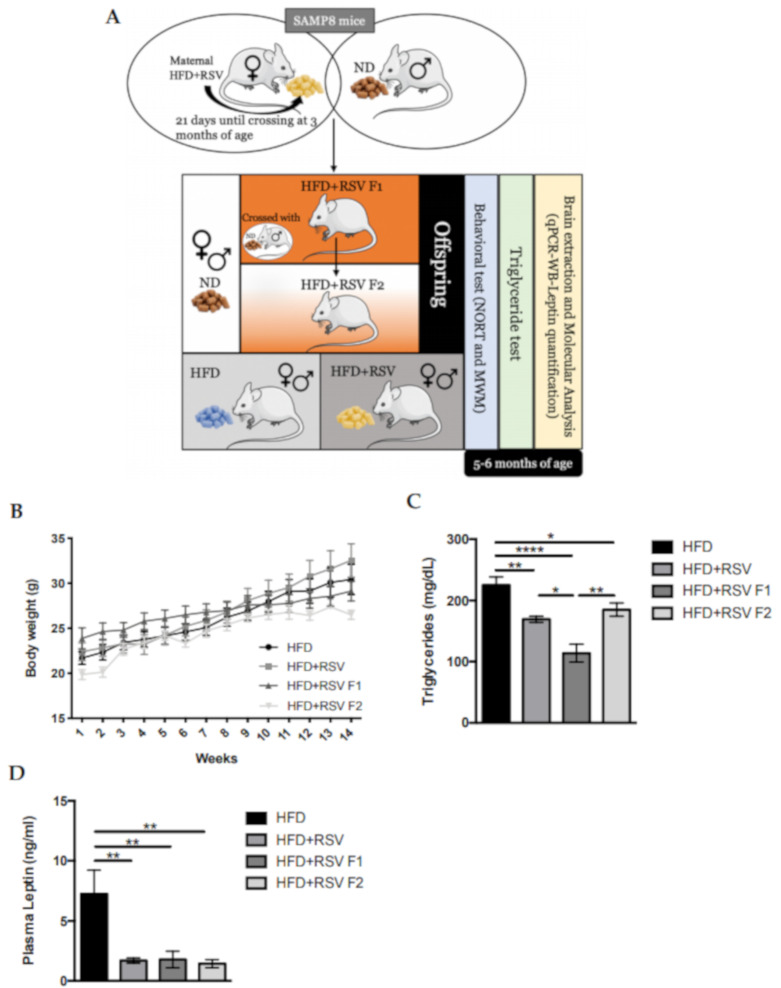
Overview of the experimental design and procedure to obtain the offspring of HFD+RSV (direct exposed parental group), HFD + RSV F1 (direct exposed generation—intergenerational inheritance), HFD + RSV F2 (indirect exposed generation - transgenerational inheritance). Mice performed the behavioral test that measured memory state, later euthanized, and brain extraction was performed for the subsequent molecular analysis at 6 months of age (**A**). Results of body weight from each week (**B**). Results of TG (mg/dL) in blood (**C**). Results of plasma leptin levels (ng/mL) (**D**). Values represented are mean ± Standard error of the mean (SEM); *n* = 60 (HFD *n* = 14, HFD + RSV *n* = 15, HFD + RSV F1 *n* = 14, HFD + RSV F2 *n* = 15; for each group). Statistics: * *p* < 0.05; ** *p* < 0.01; **** *p* < 0.0001.

Body weight evolution was measured starting from 1 month of age, and was then monitored for 14 weeks. All mice groups increased body weight along with the experiment. Furthermore, no differences between HFD and HFD + RSV groups were found (Figure 1B). Next, we measured the triglycerides (TG) and leptin plasma levels of SAMP8 mice to determine the induction of the MeS after HFD. We found a significant decreased TG levels in HFD SAMP8 mice directly supplemented with RSV (HFD +R SV group), and their offspring, compared to HFD mice in F1 (Figure 1C). Remarkably, we found a significant decrease in leptin plasma levels in HFD+RSV groups in comparison with the HFD group. (Figure 1D).

### 2.2. RSV Prevented Cognitive Impairment Induced by HFD in SAMP8 Mice across Generations

**Figure 2:**
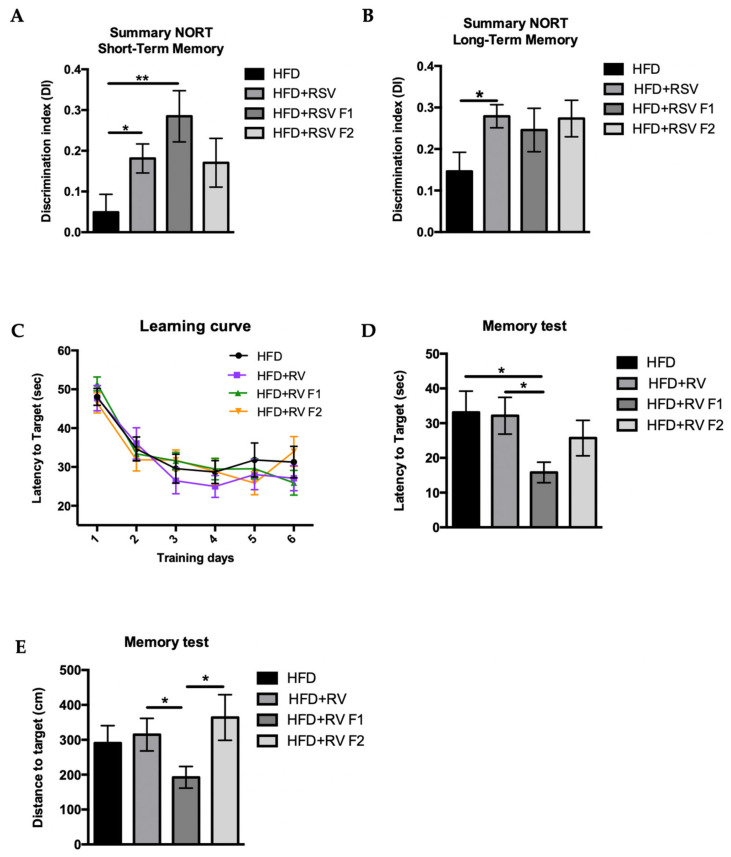
Results of Discrimination index (DI) of short-term memory (**A**) and long-term memory (**B**) from NORT of SAMP8 at 6 months of age for all. Results of Morris Water Maze (MWM) in SAMP8 mice at 6 months of age for all groups. The learning curve of the memory test for 6 days (**C**). Latency to the target in time at day 7 (spatial memory test) (**D**), and distance traveled at day 7 (spatial memory test) (**E**). Values represented are mean ± Standard error of the mean (SEM); *n* = 60 (HFD *n* = 14, HFD + RSV *n* = 17, HFD + RSV F1 *n* = 14, HFD + RSV F2 *n* = 15; for each group, same sample size of females and males). Statistics: * *p* < 0.05; ** *p* < 0.01.

Mice cognitive state was evaluated by the novel object recognition test (NORT) and morris water maze (MWM). The HFD group showed an impaired short-term memory in comparison with HFD+RSV and HFD + RSV F1 groups. Also, we found an improvement of discrimination index (DI) between the HFD and HFD + RSV F2 group, but a lower extend than for F1, demonstrating the efficacy of the RSV dietary supplementation, including across generations (Figure 2A). Furthermore, a clear improvement in the long-term memory in all HDF+RSV groups was found, being only significantly increased in the HDF + RSV directly exposed parental group (Figure 2B). We also found an improvement in cognition regarding the spatial memory tested by MWM. Namely, every mouse reduced the latency to platform on the test day in comparison with the first day of learning with no effect of RSV supplementation (Figure 2C). However, we observed that the HFD + RSV F1 group showed a significant decrease in the latency to reach the target compared to the other groups (Figure 2D). Likewise, the HFD + RSV F1 was the unique group that had a significant decrease in distance to the target, confirming the RSV’s effect on cognition in the offspring (Figure 2E).

### 2.3. Adult Offspring of SAMP8 Mice Showed Global Changes in Methylation Patterns and Its Machinery after Maternal HFD + RSV Diet, as Well as a Correlation with Cognitive Improvement

**Figure 3:**
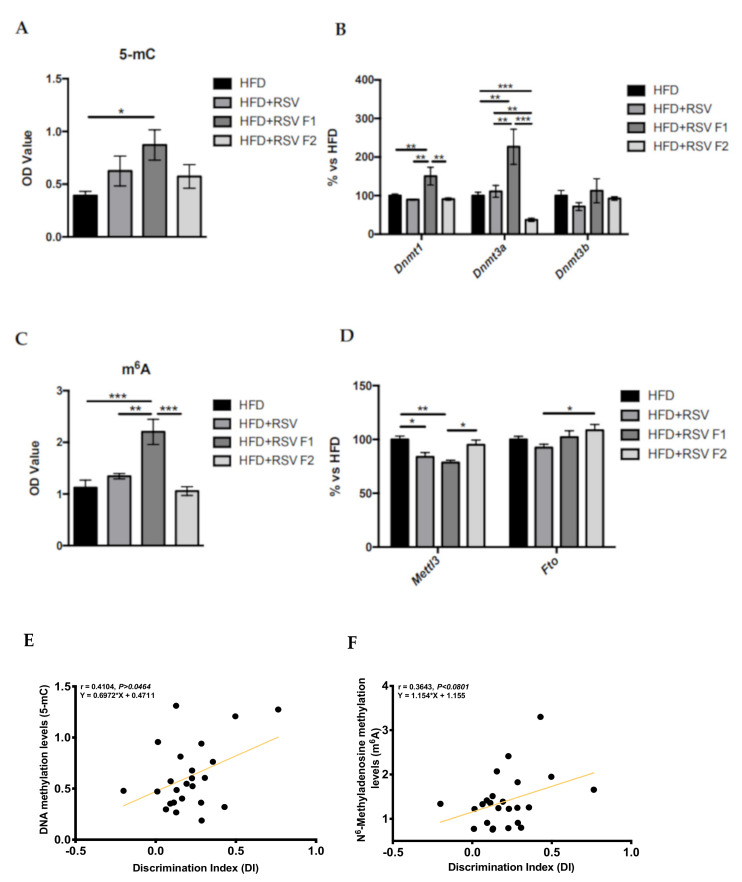
5-mC levels in the hippocampus of SAMP8 mice at 6 months of age for all groups (**A**). Relative gene expression of *Dnmt1, Dnmt3a, and Dnmt3b* (**B**). m^6^A levels from the hippocampus of SAMP8 mice at 6 months of age for all groups (**C**). Relative gene expression for Mettl3 and Fto (**D**). Correlations between 5-mC levels (**E**), m^6^A (**F**) with discrimination index (DI). Gene expression levels were measured by real-time PCR from hippocampal tissue. Gene expression data from each RSV group were compared to the HFD group (set at 100%); *n* = 16-24 (HFD *n* = 4-6, HFD + RSV *n* = 4-6, HFD + RSV F1 *n* = 4-6, HFD + RSV F2 *n* = 4–6; for each group, females *n* = 3–4, males *n* = 3–4). Statistics: * *p* < 0.05; ** *p* < 0.01; *** *p* < 0.001. Person correlations were performed between the behavioral test and molecular parameters for each group (*n* = 6). R and p-values were indicated on graphs. The level of significance was *p* < 0.05.

We studied the 5-mC and m^6^A levels in the hippocampus of SAMP8 mice at 6 months of age after the HFD exposure and RSV supplementation. On one hand, we only found that 5-mC levels were significantly increased in HFD + RSV F1 mice compared to the HFD group (Figure 3A). In the same way, we studied the gene expression of several methyltransferases, which modulate this epigenetic mark (Figure 3B). Results showed a significantly increased gene expression of *DNA methyltransferase 1 (Dnmt1)* and *DNA methyltransferase 3 alpha (Dnmt3a)* in the HFD + RSV F1 group in comparison with the other groups, confirming the epigenetic inheritance in the next generation directly exposed to RSV and losing the pattern maintenance in the indirectly exposed F2 generation (Figure 3B). The m^6^A levels were then measured. We found that the HFD + RSV F1 group showed significantly increased levels compared to the other groups (Figure 3C). In parallel, there was a significantly reduced gene expression of the enzymes *Methyltransferase like 3 (Mettl3)* and *FTO alpha-ketoglutarate dependent dioxygenase (Fto)* in HFD + RSV and HFD + RSV F1 compared to the HFD group (Figure 3D), as well as increased m^6^A levels in the HFD + RSV F2 group in comparison with the HFD + RSV F1. Next, we only found a significantly increased gene expression of *Fto* in the HFD + RSV F2 group (Figure 3D). By last, we found a positive correlation between 5-mC levels and the DI evaluated in NORT [*r* = 0.6972, *p* = 0.0464], showing that increased 5-mC levels is associated with cognitive improvement (Figure 3E). Likewise, a clear tendency of a positive correlation between m^6^A levels and the DI was evaluated [*r^2^* = 0.3643, *p* = 0.0801], suggesting that increased m6A levels are associated with cognitive improvement (Figure 3F).

### 2.4. RSV Diet Modified the Inflammatory Markers in the Hippocampus of SAMP8 and Its Offspring under HFD

**Figure 4:**
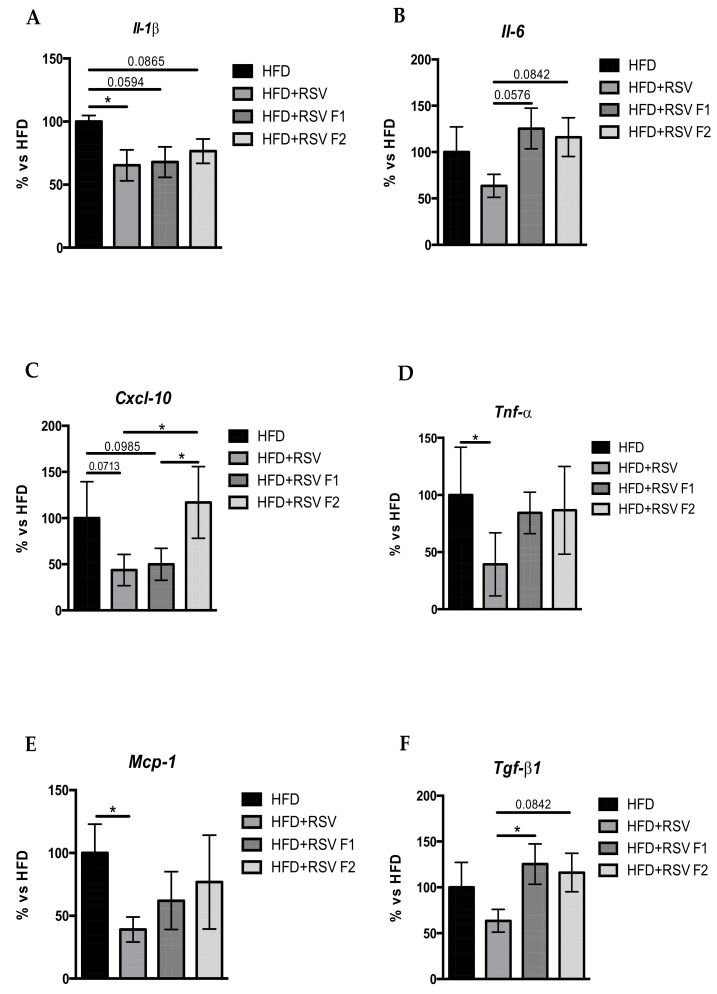
Results of gene expression of inflammatory markers for *Il1-ß* (**A**), *Il-6* (**B**), *Cxcl-10* (**C**), *Tnf-α* (**D**), *Mcp-1* (**E**), and *Tgf-ß1* (**F**) in the hippocampus of SAMP8 mice at 6 months of age. Gene expression levels were measured by real-time PCR from hippocampal tissue. Gene expression data from each RSV group were compared to the HFD group (set at 100%); *n* = 16-24 (HFD *n* = 4-6, HFD + RSV *n* = 4–6, HFD + RSV F1 *n* = 4–6, HFD + RSV F2 *n* = 4–6; for each group, females *n* = 3–4, males *n* = 3–4). Statistics: * *p* < 0.05.

One of the main effects of RSV is its anti-inflammatory effect. For this reason, we studied the gene expression of some pro-inflammatory markers in the hippocampus of SAMP8 mice. Firstly, we found a significantly decreased gene expression for *Il1-ß*, *Il-6*, *C-X-C motif chemokine ligand 10 (Cxcl-10)*, *the pro-inflammatory factors monocyte chemoattractant protein 1 (Mcp-1)*, *Transforming growth factor beta 1 (Tgf-ß1)*, and a significantly decreased for *Tnf-α* in the HFD + RSV compared to the HFD group (Figure 4A–F). Moreover, the HFD + RSV F1 and HFD + RSV F2 groups showed mainly the same gene expression pattern for pro-inflammatory markers *Il1-ß*, *Il-6,* and *Mcp-1* and *Tgf-ß1* suggesting the multigenerational effects of RSV. However, *Cxcl-10* gene expression changes were lost in the F2 group (Figure 4C).

### 2.5. RSV Diet Rescued Synaptic Dysfunction, Reverting the Effects of HFD in the Hippocampus of SAMP8 Mice and Its Offspring

**Figure 5:**
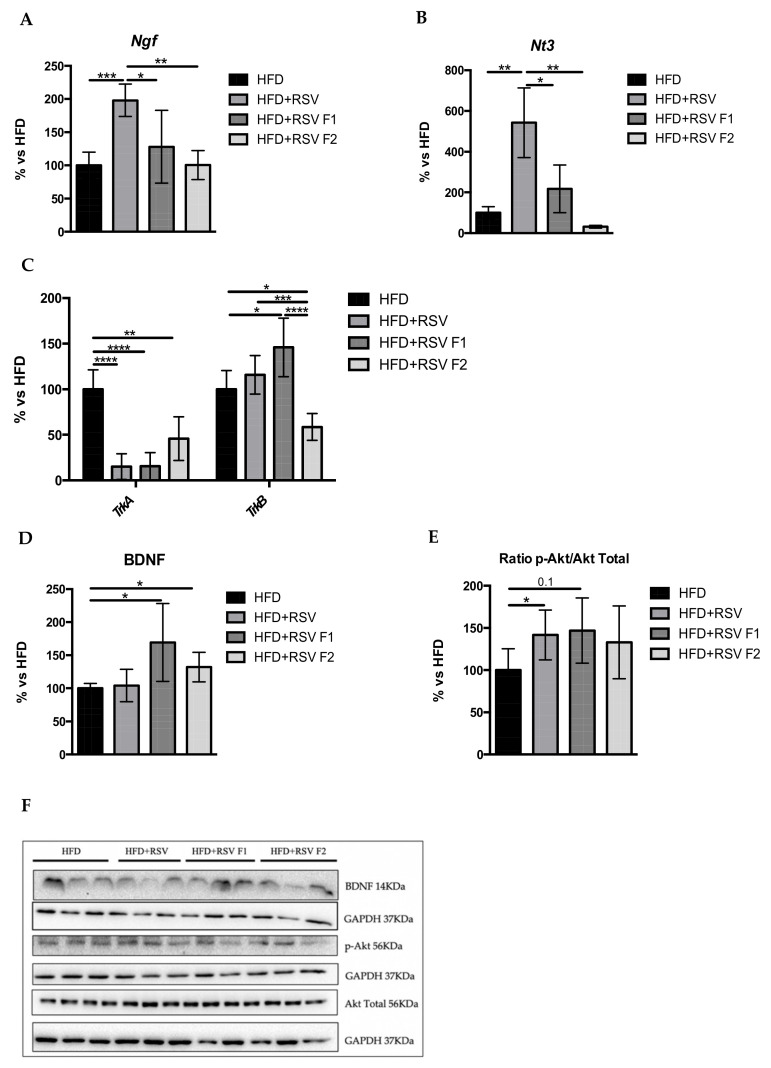
Synaptic plasticity markers in the hippocampus of SAMP8 mice at 6 months of age. Results of gene expression of *Ngf* (**A**), *Nt3* (**B**), and their receptors *TrkA* and *TrkB* (**C**). Quantifications (**D**,**E**) and representative results by WB of BDNF and p-Akt (**F**). Gene expression levels were measured by real-time PCR from hippocampal tissue. Data from each group were compared to the HFD group (set at 100%). Mean ± Standard error of the mean (SEM) in bar graphs are adjusted to 100% for each gene of HFD group; *n* = 16–24 (HFD *n* = 4–6, HFD + RSV *n* = 4–6, HFD + RSV F1 *n* = 4–6, HFD + RSV F2 *n* = 4–6; for each group, females *n* = 3–4, males *n* = 3–4). Statistics: * *p* < 0.05; ** *p* < 0.01; *** *p* < 0.001; **** *p* < 0.0001.

To evaluate the synaptic dysfunction, we investigated several neurotrophins that modulate this process. Our findings were that the HFD + RSV group showed a statistically significant increased gene expression in neurotrophins such as *Nerve growth factor (Ngf)* and *Neurotrophin-3 (Nt3*) in comparison with the other groups (Figure 5A,B). Likewise, we evaluated the gene expression levels of its receptors, and we found that the *Neurotrophic receptor tyrosine kinase 2 (TrkB)* had a significant increase in the HFD+RSV F1 group in comparison with the other groups (Figure 5C). However, we obtained a significant decrease in gene expression of *Neurotrophic receptor tyrosine kinase 1 (TrkA)* (Figure 5C) despite the increased *Ngf* expression levels. Next, the WB analysis revealed a significant increase in BDNF protein levels in the HFD+RSV F1, but no change was found in the HFD+RSV (directly exposed parental group) and HFD+RSV F2 (indirectly exposed generation) groups compared to the HFD group (Figure 5D). Moreover, the ratio of phosphorylated protein kinase B (p-Akt) and total protein levels (p-Akt/Akt total) was measured, and we found a significant increase in the HFD + RSV group in comparison with HFD as well as a tendency to maintain the same pattern protein levels of p-Akt in the next generation, suggesting, in this case, the intergenerational inheritance of RSV (Figure 5E).

## 3. Discussion

The global increase in life expectancy can also be associated with cognitive impairment and AD through lifestyle changes [41]. The nutrition as a key factor involved in cognitive decline onset is clear [42]. Indeed animal and human studies have highlighted the link between alterations in life environment and increased risk of cognitive decline in later life, including effects on offspring [43,44]. A growing body of evidence demonstrates that HFD promotes MeS associated with impaired glucose tolerance, hypertriglyceridemia, increased leptin levels, among others, being relevant for the progression of age-related cognitive decline and AD [45,46]. However, although some knowledge exists the mechanisms by which environmental changes can have long-term effects on offspring are relatively unclear. To date, no definitive mechanisms have been demonstrated that could explain multigenerational inheritance after diet or supplementation exposure. However, there have been reports of epigenetic modification following HFD intervention within the next generation, including RSV interventions. Thus, in the present study, we showed that maternal RSV supplementation not only recovers some of the key events presented in neurodegeneration such as epigenetic modifications, neuroinflammation and synaptic dysfunction as well as cognitive impairment, but we also demonstrated its effects on offspring. Our previous data demonstrated that RSV intake supplementation promoted changes in the cognitive state and molecular pathways in the hippocampus of SAMP8 transmitted to offspring [32].

On the one hand, as mentioned, HFD is considered as a main cause to the onset of MeS in animal models. Importantly, SAMP8 mice have a particular response to HFD. For instance, weight gain or glucose tolerance was not impaired after HFD, probably because SAMP8 demonstrates increased insulin sensitivity in normal diet conditions [47]. On the other hand, it is well described that maternal HFD sensitizes offspring to metabolic dysregulation and that RSV dietary supplementation improved most of the altered metabolic dysregulation [48]. Here, we reported that body weight is unchanged among groups, suggesting the same effect of the HFD across generations, and confirming the previous studies in which we did not observe changes in body weight and caloric intake in SAMP8 fed with HFD or HFD+RSV [49,50]. Interestingly, we found that RSV alleviates some MeS traits induced by HFD by reducing triglycerides and leptin plasma levels in all HFD+RSV groups compared to the HFD group, suggesting that RSV can promote peripheral effects regarding hypertriglyceridemia and leptin resistance induced by HFD. Furthermore, a recent report described that an RSV diet after weaning could alleviate leptin resistance induced by HFD [51].

Considering the well-demonstrated beneficial effects of RSV regarding cognition [32], we examined cognition by NORT and MWM. Results revealed HFD-fed mice supplemented with RSV displayed better cognitive performance than mice not supplemented, showing higher DI in short- and long-term memories. The novelty was identified for F1 and F2 mice after 24 h of the first trial, indicating a beneficial effect of maternal RSV in offspring, albeit not direct RSV contact with individuals. In spatial memory analysis, some discrepancies were found among HFD+RSV groups, and although a better spatial memory was determined in the F1 generation, these improvements disappear in the F2 generation.

As mentioned, RSV has pleiotropic actions in the organism modifying several molecular pathways starting with its antioxidant or anti-inflammatory actions, to the activation of NAD-dependent protein deacetylase sirtuin-1 (SIRT1), thus RSV can affect epigenetic marks [52,53,54,55,56]. Regarding the intervention with HFD, RSV protective role against HFD has been described [57,58] as well as its neuroprotective effects which can be transmitted across generations by epigenetic modifications in parents [59,60]. In this regard, in addition to improvements in cognition, changes in epigenetic patterns induced by RSV have been found, including in their offspring. It has been demonstrated that5-mC is essential for learning and memory, cognitive function [28], age-related alterations, as well as synaptic plasticity [61]. Remarkably, we found a higher degree of 5-mC levels in all HFD+RSV groups, being statistically significant in the F1 generation. Accordingly, we showed changes in the methylation enzymatic machinery (*Dnmt1* and *Dnmt3a*) gene expression profile in the HFD + RSV F1 group, suggesting these two enzymes’ requirements to promote changes in 5-mC pattern modifications. Similarly, several reports have shown the modification of 5-mC patterns in offspring after maternal supplementation of other compounds, such as folate [62], methionine [63], choline [64] or restrictive diet [65]. Likewise, in our study, we found a significant correlation between the increased 5-mC levels and cognitive improvement, suggesting the association of these two parameters in SAMP8. Hence, these reports and our results pointed out the 5-mC as a potential epigenetic target for cognitive decline in ageing and AD onset. Next, we evaluated the m^6^A an epigenetic mark, the most abundant modification in eukaryotic RNA. m^6^A RNA methylation and gene expression changes are determined in development and related to AD onset [66,67]. Nevertheless, few studies focused on the association between m^6^A and cognitive impairment and, until now, no in vivo study has demonstrated the participation of the m^6^A in cognitive decline, in aging and AD. In this regard, abnormalities in m^6^A levels, including the *Mettl3* and *Fto* gene expression, were reported in the cortex and hippocampus of double transgenic mice of amyloid precursor protein and presenilin 1 (APP/PS1) mice, a model of AD, in comparison with the healthy control [66]. Thus, our results provide the first evidence in which RSV consumption induced changes in the hippocampus of SAMP8, a model of accelerated senescence and early-onset AD, in offspring for this epigenetic marker. Strikingly, the highest levels of m^6^A appear in the HFD + RSV F1 group, but this epigenetic mark was lost in F2. These results can be explained, in part, by underlying germline effects promoted by RSV. Those effects were not found in the F2 generation due to is the indirectly RSV exposed generation group. As it is well-known, some epigenetic marks are erased in each generation. In parallel, the expression of *Mettl3* in the hippocampus of HFD+RSV in the F1 generation was lower than the HFD group and HFD + RSV F2, suggesting intergenerational inheritance, but not transgenerational effect. In the *Fto* expression case, we only found changes between the HFD+RSV and HFD + RSV F2 groups, confirming the loss of the inheritance effect promoted by RSV in the hippocampus of SAMP8 for this epigenetic mark. Furthermore, we found a tendency to correlate the m^6^A levels and cognitive improvement, suggesting the participation of this epigenetic mark in cognition.

Neuroinflammation has been linked with the risk of developing cognitive impairment [68,69]. At the same time, the anti-inflammatory effect of RSV by different mechanisms such as SIRT1 activation, nuclear factor kappa-light-chain-enhancer of activated B cells (NF-kß) down-regulation, including the modulation of microglial activation, has been well established, and promotes a reduction of pro-inflammatory cytokines already mentioned [70,71]. A diminished gene expression of several pro-inflammatory cytokines in HFD + RSV groups was found in the current work, demonstrating transmission of the anti-inflammatory effect across generation. However, the HFD + RSV F2 group’s anti-inflammatory pattern was bizarre (inconsistent), suggesting low transmission across generations (transgenerational epigenetic inheritance), losing beneficial RSV effect when undirect action was evaluated. Indeed, previous findings which demonstrate the anti-inflammatory effects induced by maternal RSV supplementation showed the same discrepancies regarding its transgenerational effect [32]. The fact that inflammation and oxidative stress processes can be activated in different ways could explain the loss of effect in offspring [72,73,74].

Given that synaptic disruption is the higher correlate of cognitive loss, alterations in the synaptic plasticity process are considered one of the most critical mechanisms of age-related cognitive decline [75] and AD [76]. Thus, the role of neurotrophins in the impaired memory formation and neuronal degeneration is well known [77,78]. For instance, long term potentiation (LTP) is necessary for memory formation and is maintained by BDNF and NT3 at TrkB receptor [77], resulting in structural changes at the synapse [78]. Likewise, NGF and its neurotrophin receptor TrkA promote the same effect in synaptic contact between neurons [79]. In this study, we found robust changes for some of the neurotrophins and receptors in the HFD + RSV groups. In line with our results, some studies have described that RSV induces the expression of glial cell-derived neurotrophic factor (GDNF), BDNF, and NT3 under different interventions in the brain, confirming our data [80,81,82]. Moreover, little has been published on transgenerational changes in neurotrophins after maternal RSV supplementation. Here, we found subtle changes in the HFD + RSV F1 and HFD + RSV F2 groups, demonstrating the reduced capacity of RSV to modify epigenetic marks that are maintained in the indirectly exposed generation, and consequently facilitating changes in the expression of neurotrophic pathways across generations, which are related to molecular neuroprotective effects observed in F1 and F2. The increased p-Akt ratio in HFD+RSV groups in comparison with the HFD group was found, confirming the neuroprotective effect of RSV by promoting synaptic plasticity.

**Figure 6:**
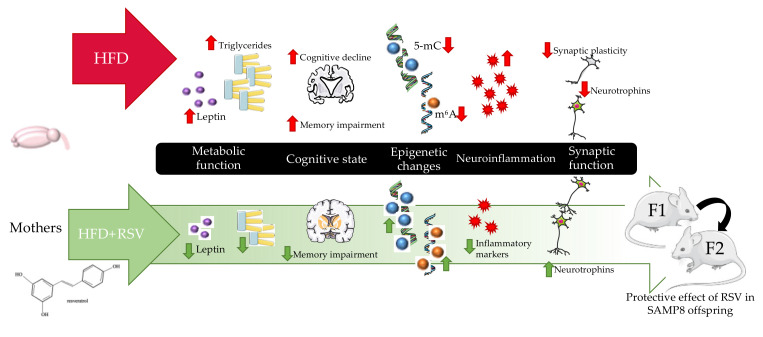
Schematic representation of the neuroprotective effects observed of maternal RSV intake under HFD across generations in SAMP8.

Taken together, the new data presented above demonstrated that maternal RSV supplementation mediated several modifications under HFD through reduction of hypertriglyceridemia and leptin resistance, epigenetic alterations, neuroinflammation, and synaptic dysfunction for maintaining better cognitive performance in SAMP8 and their offspring (Figure 6). Therefore, this study shows new important insights of the beneficial effects and mechanisms promoted by RSV after maternal supplementation on inheritance, reinforcing the importance of lifestyle interventions in preventing cognitive decline and neurodegenerative diseases through strategies based on a healthy diet or food intake supplementation.

## 4. Materials and Methods

### 4.1. Animals

SAMP8 offspring were generated from the high-fat diet (HFD) group (Females F0 (HFD F0, *n* = 4) that had access to the diet (AIN-93G) which wasmodified to provide 60% of Kcal from fat with carbohydrate/protein/fat ratio of 16:23:61 % of Kcal). Specifically, HFD + RSV contained 1g/kg w/w of RSV (Females HFD+RSV F0, *n* = 4). Mice were fed for 2 months and the supplementation diet was interrupted before crossing HFD + RSV F0 females with ND males, who had access to standard chow with carbohydrate/protein/fat ratio of 64:19:17 % of Kcal to obtain the first generation (the intergenerational inheritance offspring (HFD + RSV F1, *n* = 14; females *n* = 4, males *n* = 10) for accelerating the transgenerational effect one generation early (in F2 instead of F3). Then, we crossed HFD + RSV F1 females (Females HFD + RSV F1, *n* = 4) with ND males to generate the second generation (the transgenerational inheritance offspring HFD + RSV F2, *n* = 15; females *n* = 10, males *n* = 5). HFD + RSV F1 and HFD + RSV F2 groups were fed a standard diet (Figure 1A). The intervention sample size was chosen following previous studies in our laboratory and using one of the available interactive tools (http://www.biomath.info/power/index.html). Animals had free access to food and water and were kept under standard temperature conditions (22 ± 2 °C) as well as 12 h:12 h light-dark cycles (300 lux/0 lux) until 6 months of age. The body weight of the animals was monitored weekly.

Mice were treated according to the European Community Council Directive 86/609/EEC and were approved by the Institutional Animal Care and Use Committee of the University of Barcelona (670/14/8102, approved at 11/14/2014) and by Generalitat de Catalunya, Spain (10291, approved at 1/28/2018). Every effort was made to minimize animal suffering and to reduce the number of animals.

### 4.2. Behavioral Test

#### 4.2.1. Novel Object Recognition Test (NORT)

In summary, mice were placed in a 90°, two-arms, 25 cm-long, 20 cm-high, 5 cm-wide black maze. Firstly, mice were individually habituated to the maze for 10 min for 3 days. On the fourth day, the animals were exposed to a 10 min acquisition trial (first trial), during which they were placed in the maze in the presence of two identical novel objects at the end of each arm. After 2 h, the animal was exposed to two objects, in this time, one old object and one novel object. On the fifth day, the animal was exposed for 24 h in the same conditions. The time that mice explored the Novel object (TN) and Time that mice explored the Old object (TO) were measured generating a Discrimination Index (DI) that we calculated as (TN − TO)/(TN + TO) in both performances evaluating short- and long-term memory of animals. To avoid object preference biases objects were counterbalanced. The maze, surface, and objects were cleaned with 70% ethanol between the animal trials to eliminate olfactory cues.

#### 4.2.2. Morris Water Maze (MWM)

To evaluate mice’s spatial learning and memory, we exposed the animals to an open circular pool (100 cm in diameter, 50 cm in height) filled with water. Water was painted white with latex in order to opaque it, and its temperature was 22 ± 1 °C. Two perpendicular axes were defined; thus, the water surface was divided into four quadrants (NE, SE, SW, and NW) and five starting points were set (NE, E, SE, S, and SW). Four visual clues (N, S, E, and W) were placed on the walls of the tank so that the animal could orientate and fulfill the objective. MWM consisted of training mice to find a submerged platform (Learning phase), for 6 consecutive days, and assess whether the animal had learned and remembered where the platform was on the last day (day 7) when it was removed (Spatial memory test). For 6 learning days, 5 trials were performed in 5 different points that we explained before. In each trial, the mouse was placed gently into the water, facing the wall of the pool, allowed to swim for 60 s, and there was no a resting time between trials. If the animal could not locate the platform, the investigator guided it to the platform and where it was allowed to rest and orientate for 30 s. The platform was placed approximately in the middle of one of the quadrants, 1.5 cm below the water level. Above the pool, a camera recorded the animal’s swimming paths with SMART^®^ program ver.3.0 (Panlab, Cornellà, BCN, Spain). During the learning phase, a learning curve was drawn, which represented the latency to find the platform every training day. More parameters were measured on the test day, such as the target crossings, the swim distance to target or time in the platform zone.

### 4.3. Immunodetection Experiments

#### 4.3.1. Brain Processing

Mice were euthanized one day after behavioral tests finished by cervical dislocation. Brains were immediately removed, and the hippocampus was isolated and frozen on powdered dry ice. They were kept at −80 °C until use.

#### 4.3.2. Plasma Isolated, Leptin and Triglycerides Quantification

Briefly, trunk blood was collected when the mice were euthanized using heparinized tubes (Micro tube 1.1 mL Z-Gel; Sarstedt AG & Co. KG). Plasma was isolated by centrifugation for 5 min at 10,000× *g* at 20 °C and frozen at −20 °C until assay. We quantified Leptin levels in plasma samples with the kit mouse leptin ELISA Kit (Cat. # EZML-82-K© 2013 EMD Millipore, Burlington, MA, USA). This kit to detects leptin in samples by detecting specific antibodies that bind to leptin and reacts in sufficient substrate presence. The enzyme activity was measured spectrophotometrically by colorimetric reaction at the absorbance of 450 nm and corrected by 590 nm. Therefore, the increase in absorbance is directly proportional to the amount of captured leptin in samples. Later, we quantified using a standard curve of known concentrations of mouse leptin (0.23–30 ng/mL). The determination of triglycerides concentration was performed using a triglyceride meter device (Accutrend^®^ Plus, Cobas, Roche), in which a drop of blood covered the corresponding strips.

#### 4.3.3. Western Blotting (WB)

The hippocampus tissue from each animal was homogenized in lysis buffer (Tris HCl pH 7.4 mM, NaCl 150mM, EDTA 5mM and 1X-Triton X-100) containing phosphatase and protease inhibitors (Cocktail II, Sigma-Aldrich, St. Louis, MO, USA) to obtain total protein homogenates. The Bradford technique determined total protein concentration. Aliquots of 15 μg of total hippocampal protein were used and separated by sodium dodecyl sulfate-poly-acrylamide gel electrolysis (SDS-PAGE) (8–20%) and transferred into polyvinylidene difluoride (PVDF) membranes (Millipore, Burlington, MA, USA) for 2 h. The membranes were blocked in 5% non-fat milk in TRIS-buffered saline (TBS) containing 0.1% Tween 20 (TBS-T) for 1h at room temperature. Next, overnight incubation at 4 °C with primary antibodies listed in Table S1 was carried out. The following day, membranes were washed with TBS-T 3 times for 5 min and incubated with secondary antibodies for 1h at room temperature. Immunoreactive protein was viewed with a chemiluminiscence-based detection kit, following the manufacturer’s protocol (ECL Kit, Millipore, Burlington, MA, USA), and digital images were acquired using a ChemiDoc XRS+ System (BioRad, Hercules, CA, USA). Semi-quantitative analyses were done using ImageLab Software (BioRad), and results were expressed in Arbitrary Units (AU) considering control protein levels as 100%. Protein loading was routinely monitored by immunodetection of glyceraldehyde-3-phosphate dehydrogenase (GADPH).

### 4.4. RNA Extraction and Gene Expression Determination by q-PCR

Total RNA isolation from hippocampal tissue was carried out using TRIsure^TM^ reagent following the manufacturer’s instructions (Bioline Reagent, Memphis, TN, USA). The yield, purity, and quality of RNA were determined spectrophotometrically with a NanoDrop™ ND-1000 (Thermo Scientific, Waltham, MA, USA) apparatus and an Agilent 2100B Bioanalyzer (Agilent Technologies, Santa Clara, CA, USA). RNAs with 260/280 ratios and RIN higher than 7.5, respectively, were selected. Reverse transcription-polymerase chain reaction (RT-PCR) was performed as follows: 2 μg of messenger RNA (mRNA) was reverse-transcribed using the High-capacity cDNA reverse transcription Kit (Applied Biosystems, Foster City, CA, USA). Real-time quantitative PCR (qPCR) was used to quantify mRNA expression of chromatin-modifying, inflammatory, and synaptic plasticity genes listed in Table S2.

Real-time PCR was performed by using Step One Plus Detection System (Applied-Biosystems,) employing SYBR^®^ Green PCR Master Mix (Applied-Biosystems). Each reaction mixture contained 6.75 μL of complementary DNA (cDNA) (which concentration was 2 μg/μL), 0.75 μL of each primer (which concentration was 100 nM), and 6.75 μL of SYBR^®^ Green PCR Master Mix (2X).

Data were analyzed utilizing the comparative Cycle threshold (Ct) method (ΔΔCt), where the housekeeping gene level was used to normalize differences in sample loading and preparation. Normalization of expression levels was performed with β-actin for SYBR^®^ Green-based real-time PCR results. Each sample was analyzed in duplicate, and the results represent the n-fold difference of the transcript levels among different groups.

### 4.5. Global DNA Methylation Determination

Briefly, isolation of genomic DNA from hippocampal tissue was conducted using the FitAmpTM Blood and Cultured Cell DNA Extraction Kit, according to the manufacturer’s instructions. Then, Methylflash Methylated DNA Quantification Kit (Epigentek, Farmingdale, NY, USA) was used to detect methylated DNA. Specifically, this kit is based on the antibody detection of 5-mC residues that allows colorimetric quantification by reading absorbance at 450 nm using a Microplate Photometer. The absolute amount of methylated DNA (proportional to the Optical Density [OD] intensity) was measured and quantified using a standard curve plotting OD values vs. five serial dilutions of a control methylated DNA (0.5–10 ng).

### 4.6. m^6^A RNA Methylation Quantification

To determine m^6^A RNA methylation status from hippocampal tissue, we used the EpiQuik™ m^6^A Quantification Kit according to the manufacturer’s instructions (Epigentek, Farmingdale, NY, USA) using total RNA isolated from mice. The kit is based on specific antibody detection of m^6^A residues, which trigger an ELISA-like reaction that allows colorimetric quantification by reading absorbance at 450 nm using a Microplate Photometer. The absolute amount of m^6^A (proportional to the Optical Density [OD] intensity) was measured and quantified using a standard curve plotting OD values vs. six serial dilutions of a control m^6^A (0.01–0.5 ng).

### 4.7. Data Analysis

The statistical analysis was conducted using GraphPad Prism ver. 8 statistical software. Data are expressed as the mean ± Standard Error of the Mean (SEM) of at least 7 samples per group for behavioral tests and 3–5 samples per group for molecular analysis. Means were compared by One-way Analysis of variance (ANOVA), followed by Tukey post-hoc analysis or two-tail Student’s t-test when it was necessary. Correlations among different parameters were analyzed with Pearson’s correlation. Statistical significance was considered when p-values were <0.05. The Statistical outliers were determined with Grubbs’ test and subsequently removed from the analysis.

## Supplementary Materials

Supplementary materials can be found at https://www.mdpi.com/1422-0067/22/3/1453/s1. Table S1. Antibodies used in Western blot studies. Table S2. Primers used in qPCR.

## Abbreviations

AD	Alzheimer’s disease
Akt	Protein kinase B
ANOVA	One-way analysis of variance
APP/PS1 mice	Double transgenic mice of amyloid precursor protein and presenilin 1
BDNF	Bran-derived neurotrophic factor
cDNA	Complementary DNA
CNS	Central nervous system
Ct	Cycle threshold
Cxcl-10	C-X-C motif chemokine ligand 10
DI	Discrimination index
DNA	Desoxyribonucleic acid
5-hmC	Hydroxymethylation
5-mC	DNA methylation
Dnmt1	DNA methyltransferase 1
Dnmt3a	DNA methyltransferase 3 alpha
Dnmt3b	DNA methyltransferase 3 beta
Fto	Alpha-ketoglutarate dependent dioxygenase
GAPDH	Glyceraldehyde-3-phosphate dehydrogenase
GDNF	Glial cell-derived neurotrophic factor
HFD	High fat diet
HFD+RSV	High fat diet with resveratrol parental group
HFD+RSV F1	High fat diet with resveratrol first generation
HFD+RSV F2	High fat diet with resveratrol second generation
Il-6	Interleukin 6
Il-1β	Interleukin 1 beta
LTP	Long term potentiation
m^6^A	N-6-methyladenosine
Mcp-1	Monocyte chemoattractant protein 1
MeS	Metabolic syndrome
Mettl3	Methyltransferase like 3
mRNA	Mature messenger RNA
MWM	Morris water maze
ND	Normal diet
NF-κβ	Nuclear factor NF-kappa-B
NGF	Nerve growth factor
NORT	Novel object recognition test
NT3	Neurotrophin-3
OD	Optical density
OS	Oxidative stress
p-Akt	Phosphorylated RAC-alpha serine/threonine-protein kinase
PCR	Polymerase chain reaction
PVDF	Polyvinylidene difluoride
qPCR	Real-time quantitative polymerase chain reaction
RNA	Ribonucleic acid
RT-PCR	Reverse transcriptional-polymerase chain reaction
RSV	Resveratrol
SAMP8	Senescence-accelerated mouse prone 8
SAMR1	Senescence-accelerated mouse resistant 1
SDS-PAGE	Sodium dodecyl sulfate-polyacrylamide gel electrolysis
SEM	Standard error of the mean
SIRT1	NAD-dependent protein deacetylase sirtuin-1
TBS	Tris-buffer saline
TBS-T	Tris-buffer saline tween 20
TG	Triglycerides
TGF-β1	Transforming growth factor beta 1
TN	Time with new object
TNF-α	Tumor necrosis factor-alpha
TO	Time with old object
TrkA	Neurotrophic receptor tyrosine kinase 1
TrkB	Neurotrophic receptor tyrosine kinase 2
WB	Western blot
