# Correction: Izquierdo et al. Resveratrol Supplementation Attenuates Cognitive and Molecular Alterations under Maternal High-Fat Diet Intake: Epigenetic Inheritance over Generations. *Int. J. Mol. Sci.* 2021, *22*, 1453

**DOI:** 10.3390/ijms22179155

**Published:** 2021-08-25

**Authors:** Vanesa Izquierdo, Verónica Palomera-Ávalos, Mercè Pallàs, Christian Griñán-Ferré

**Affiliations:** 1 Department of Pharmacology and Therapeutic Chemistry, Institut de Neurociències-Universitat de Barcelona, Avda. Joan XXIII, 27, 08028 Barcelona, Spain; vanessa_izquierdo@hotmail.com (V.I.); pallas@ub.edu (M.P.); 2 Department of Cellular and Molecular Biology, University Center of Biological and Agricultural Sciences, University of Guadalajara, km 15.5 Guadalajara-Nogales Highway, Zapopan 45110, Jalisco, Mexico; vpalomera@hotmail.com

The author wishes to make the following correction to this paper [1]:

In the original article, there was a mistake in Figure 5F. All WBs experiments were performed with 14 samples in total, where the 2 bands that are not shown correspond to the control group without dietary intervention, so the article shows the 12 bands that correspond to the HFD, HFD + RSV, HFD + RSV F1 and HFD + RSV F2. The corrected Figure 5F appears below.

The authors apologize for any inconvenience caused and state that the scientific conclusions are unaffected. The original article has been updated.

## Figures and Tables

**Figure 5:**
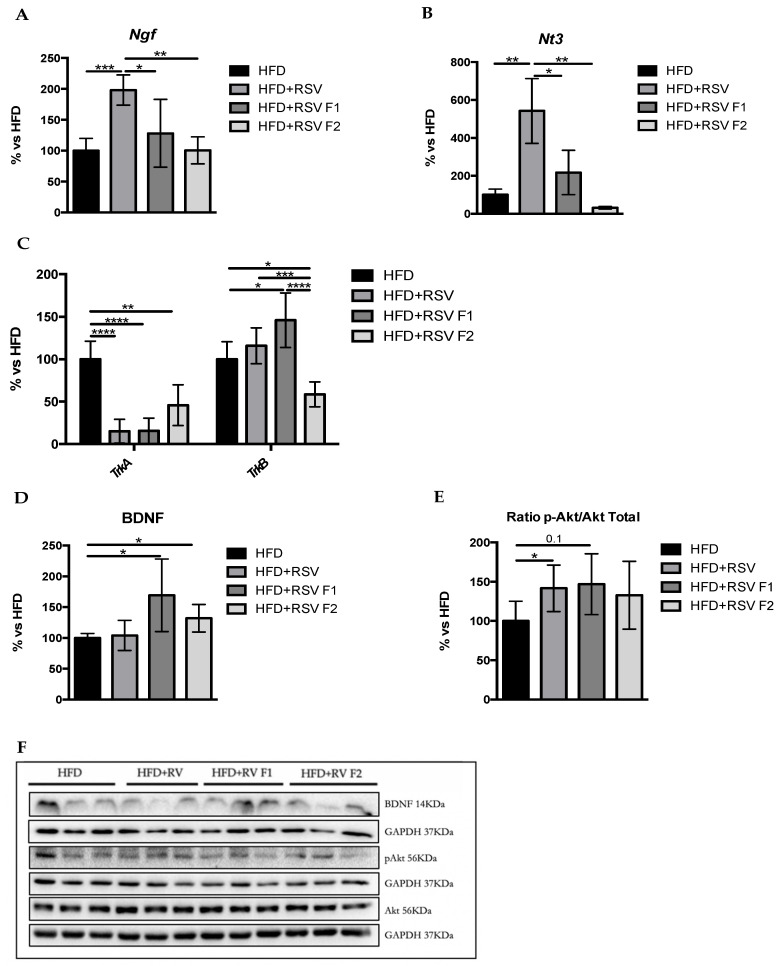
Synaptic plasticity markers in the hippocampus of SAMP8 mice at 6 months of age. Results of gene expression of *Ngf* (**A**), *Nt3* (**B**), and their receptors, TrkA and TrkB (**C**). Quantifications (**D**,**E**) and representative results by WB of BDNF and p-Akt (**F**). Gene expression levels were measured by real-time PCR from hippocampal tissue. Data from each group were compared with the HFD group (set at 100%). The means and standard error of the mean (SEM) in bar graphs are adjusted to 100% for each gene of the HFD group; *n* = 16–24 (HFD *n* = 4–6, HFD + RSV *n* = 4–6, HFD + RSV F1 *n* = 4–6, HFD + RSV F2 *n* = 4–6; for each group, females: *n* = 3–4, males: *n* = 3–4). Statistics: * *p* < 0.05; ** *p* <0.01; *** *p* < 0.001; **** *p* < 0.0001.
