# Supplementary material for: Resveratrol Supplementation Attenuates Cognitive and Molecular Alterations under Maternal High-Fat Diet Intake: Epigenetic Inheritance over Generations
Source: Int J Mol Sci. 2021 Feb 1;22(3):1453. doi: 10.3390/ijms22031453 (PMC7867164; doi:10.3390/ijms22031453)
Supplement: Supplementary file 1 [file ijms-22-01453-s001.pdf]

Table 1. Antibodies used in Western blot studies.

Antibody	Host	Source/Catalog	WB dilution
**BDNF**	Rabbit	Santa Cruz/sc-20981	1:1000
**p-Akt (S473) (D9E)**	Rabbit	Cell signaling/#4060	1:1000
**Akt**	Rabbit	Cell signaling/#9272	1:1000
**GAPDH**	Mouse	Millipore/MAB374	1:2000
**Goat-anti-mouse HRP conjugated**		BioRad/170-5047	1:2000
**Goat-anti-rabbit HRP conjugated**		BioRad/170-6515	1:2000

Table 2. Primers used in qPCR studies.

Target	Forward primer (5′-3′)	Reverse primer (5′-3′)
Cxcl-10	GGCTAGTCCTAATTGCCCTTGG	TTGTCTCAGGACCATGGCTTG
Dnmt1	ACCTGGAGAGCAGAAATGGC	TGAAAGGGTGTCACTGTCCG
Dnmt3a	GAGCCGCCTGAAGCCC	TCTTCCTTGCCACGGTTCTC
Dnmt3b	TGCCAGACCTTGGAAACCTC	GCTGGCACCCTCTTCTTCAT
Fto	GACACTTGGCTTCCTTACCTG	CTCACCACGTCCCGAAACAA
Mettl3	TTAGCATCTGGTCTGGCCTCTT	TGACCTTCTTGCTCTGCTGTTC
Mcp1	CCCACTCACCTGCTGCTACT	TCTGGACCCATTCCTTCTTG
Ngf	GGAGCGCATCGAGTGACTT	CCTCACTGCGGCCACTATAG
Nt3	CAGGGTGAAGGGGAAAACT	AGTTCGGTCATTCAGTCTCGC
Il-1β	ACAGAATATCAACCAACAAGTGATATTCTC	GATTCTTTCCTTTGAGGCCCA
Il-6	ATCCAGTTGCCTTCTTGGGACTGA	TAAGCCTCCGACTTGTGAAGTGGT
Tgf-β1	CAGTGGCTGAACCAAAGGAGACGG	CCCCGACGTTTGGGGCTGATC
Tnf-α	TCGGGGTGATCGGTCCCCAA	TGGTTTGCTACGACGTGGGCT
TrkA	CTCCTTCTCGCCAGTGGAC	TGCCCTCAGTAGGGGAAAGA
TrkB	CGTCACTTCGCCAGCCAGCAGTAG	CTATACGCCAGGCACCACTC
β-actin	CAACGAGCGGTTCCGAT	GCCACAGGTTCCATACCCA
